# Biochemical analysis of *Hyalomma dromedarii* salivary glands and gut tissues using SR-FTIR micro-spectroscopy

**DOI:** 10.1038/s41598-024-59165-6

**Published:** 2024-04-12

**Authors:** Seham H. M. Hendawy, Heba F. Alzan, Hoda S. M. Abdel-Ghany, Carlos E. Suarez, Gihan Kamel

**Affiliations:** 1 Parasitology and Animal Diseases Department, Veterinary Research Institute, National Research Centre, 33 El Buhouth St., Dokki, Giza, 12622 Egypt; 2 Tick and Tick-Borne Diseases Research Unit, Veterinary Research Institute, National Research Centre, 33 El Buhouth St., Dokki, Giza, 12622 Egypt; 3 Present Address: Department of Veterinary Microbiology and Pathology, College of Veterinary Medicine, Washington State University, Pullman, WA 99164-7040 USA; 4 Animal Disease Research Unit, United States Department of Agricultural-Agricultural Research Service, Pullman, WA USA; 5 SESAME Synchrotron (Synchrotron-light for Experimental Science and Applications in the Middle East), Allan, 19252 Jordan; 6 Department of Physics, Faculty of Science, Helwan University, Cairo, Egypt

**Keywords:** Biophysical methods, Imaging

## Abstract

Ticks are obligatory voracious blood feeders infesting diverse vertebrate hosts, that have a crucial role in the transmission of diverse pathogens that threaten human and animal health. The continuous emergence of tick-borne diseases due to combined worldwide climatic changes, human activities, and acaricide-resistant tick strains, necessitates the development of novel ameliorative tick control strategies such as vaccines. The synchrotron-based Fourier transform infrared micro-spectroscopy (SR-FTIR) is a bioanalytical microprobe capable of exploring the molecular chemistry within microstructures at a cellular or subcellular level and is considered as a nondestructive analytical approach for biological specimens. In this study, SR-FTIR analysis was able to explore a qualitative and semi-quantitative biochemical composition of gut and salivary glands of *Hyalomma dromedarii* (*H. dromedarii*) tick detecting differences in the biochemical composition of both tissues. A notable observation regarding Amide I secondary structure protein profile was the higher ratio of aggregated strands in salivary gland and beta turns in gut tissues. Regarding the lipid profile, there was a higher intensity of lipid regions in gut tissue when compared to salivary glands. This detailed information on the biochemical compositions of tick tissues could assist in selecting vaccine and/or control candidates. Altogether, these findings confirmed SR-FTIR spectroscopy as a tool for detecting differences in the biochemical composition of *H. dromedarii* salivary glands and gut tissues. This approach could potentially be extended to the analysis of other ticks that are vectors of important diseases such as babesiosis and theileriosis.

## Introduction

Ticks are obligatory hematophagous ectoparasites that are widely distributed around the world infesting diverse vertebrate hosts either human or animal^1,2^. Importantly, ticks are considered vectors for many pathogens causing serious diseases in human, domestic and wild animals. In addition, they can cause direct damage to their vertebrate hosts due to their voracious blood feeding habits, which may also result in anemia^3,4^. Among the nine hundred tick species so far identified worldwide, *Rhipicephalus* and *Hyalomma* are the most widely distributed tick genera*.* Together, these tick species are affecting directly and indirectly the economy of cattle, camels, sheep, and goat industries in tropical and subtropical countries including Egypt^5,6^. Chemical acaricides are widely used to control tick and tick-borne diseases transmission. However, the intensive use of pesticides leads to the emergence of resistant tick strains plus accumulation of undesirable toxic chemical residues in animal food products, in addition to being an environmental hazard. These pesticide pitfalls adversely impact human and animal health^2,7^. In this context, anti-tick vaccines are currently considered as an optimal alternative as an environmentally safe strategy for controlling tick and tick-borne diseases and are regarded as a promising tool for the control of ticks and tick-borne diseases. The use of vaccines, unlike chemical acaricides, poses minimal risks for selecting acaricide-resistant ticks, since point mutations that render acaricides ineffective are less likely to alter protective epitopes on tick target proteins^7,8^, although vaccine-resistant variants can also occur.

Fourier transform infrared (FTIR) micro-spectroscopy is an emerging technique for biochemical analysis of tissues and cellular materials, that also has been recently applied in many different medical research areas^9^. It also proved to be a strong bioanalytical microprobe capable of exploring molecular chemistry within microstructures at a cellular or subcellular level. The use of a synchrotron-based infrared spectroscopic investigation allows several unique advantages, among these, the brightness advantage (100–1000 orders of magnitude) compared to the conventional IR sources due to the effective small source size, collimation, in addition to the pulsed time structure and the high degree of polarization^10,11^. FTIR micro-spectroscopy can be considered as a non-destructive and economically feasible technique that could be used for predicting the biochemical structure of different tick tissues. With the high spatial resolution of a synchrotron IR source, it is possible to precisely quantify subtle spectral differences, as it is crucial to obtain high quality information about the secondary structure variations of protein^11^. To conclude, measuring chemical maps of diverse tick antigens using SR-FTIR could be a promising first step to identify, together with several analytical methods, the shared/conserved tick antigens. In addition, detection of exclusive structure and /or conformational changes that are considered as unique taxonomical features used in the characterization of different tick species and/or genus may be also assessed^11^.

SR-FTIR can generate biochemical information on major biomarkers such as proteins, lipids, carbohydrates, and nucleic acids. The definition of spectral biomarkers specific for such biomolecules can be considered as an analytical fingerprint that may be characteristic of the investigated biological cells or tissues. Besides, the simplicity of the technique to be performed on tiny samples gives a good indicator, particularly in cases where the current “gold standards” are needed^12^.

In the field of biology, FT-IR micro-spectroscopy has been used to analyze the chemical composition of mutant and wild types of *Caenorhabditis elegans* nematodes, which allow the detection of differences in the relative amounts of carbohydrates, proteins, and lipids between both types through the vibrational spectroscopic spectra^13^. Moreover, vibrational spectroscopic imaging techniques including Atomic Force Microscopy Infrared (AFM-IR) and confocal Raman microscopy, have been also used previously to discover new biomarkers for *Babesia bovis* infections which is considered one of the most threatful tick -borne diseases. Furthermore, Attenuated Total Reflectance Fourier Transform Infrared (ATR-FTIR) has been used to discriminate infected red blood cells (RBCs) samples with *B. bovis* and non-infected RBCs based on changes in the IR spectral bands in less than two minutes, excluding sample extraction and preparation^14^.

One of the most critical steps in tick tissue analysis is sample preparation and preservation especially when used with the purpose of understanding the natural biological features of the tissue. The biological materials are most frequently probed in transmission or reflection-mode upon usage of the IR microscope.

Hereby, SR-FTIR microspectroscopy was used to reveal the biochemical composition of two different types of tick tissues (salivary glands and gut) of *Hyalomma dromedarii* (*H. dromedarii),* for deep tissue characterization.

## Results and discussion

SR-FTIR microspectroscopy is considered for the first time as a versatile and fast tool to analyze comprehensive biochemical composition of salivary glands and gut of *H. dromedarii* tick tissues. This method presents accurate information about protein and lipids composition without the necessity for sample preparation or the use of special stains. Besides, the protein and lipids composition are monitored simultaneously from a single salivary gland/ and or gut to be analyzed. SR-FTIR microspectroscopic investigation was carried out on gut and salivary glands biological tissues aiming at obtaining clues about the biochemical features of both tissue types of the *H. dromedarii* tick species at the molecular level. The biomolecular composition was attempted qualitatively, as well as semi-quantitatively. For the first instance, distinctive spectral features of biological tissues were detected for both tissues with some alterations when compared to each other. Three regions of interest were examined and will be presented throughout this manuscript: protein absorption bands (amide I and amide II), lipids’ profiles, as well as the spectral region (1000–1500 cm^−1^). Peak assignment of the characteristic IR absorption bands was performed based on Barth et al.^15^, and analysis was constructed on similar studies of biological tissues at the molecular level using FTIR microspectroscopy techniques^12,13^.

### Protein spectral region (1700–1500 cm^−1^)

**Figure 1:**
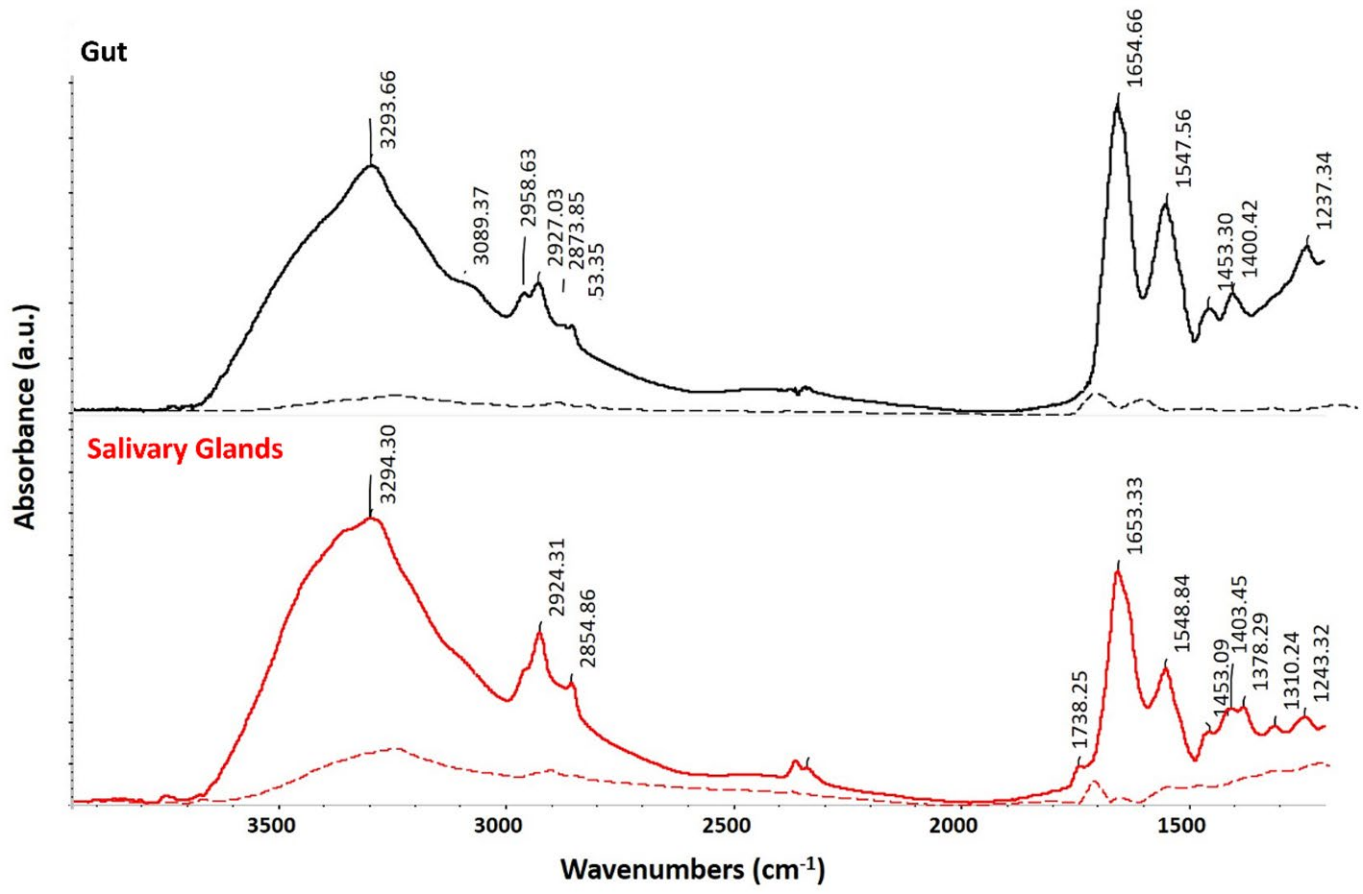
Average IR spectra of the gut and the salivary glands tissues, with their relative peak assignments. Spectra are displayed in stack mode for sake of clarity. Dashed lines represent the spectral variance for both groups of tissues.

Protein molecular vibrations were well recognized for the studied tissues comprising amide I (1700–1600 cm^−1^) which originates from the stretching vibration C=O, CN, and NH bending vibration with most intense absorption at 1650 cm^−1^, and amide II (1600–1500 cm^−1^) band which arises from the NH bending and CN stretching vibrations with minor contributions from CO bending and CC and NC stretching vibrations showed a central peak at 1550 cm^−1^^15^. The gut tick tissue sample showed distinctive an intense Amide I band at 1655 cm^−1^ and an Amide II band at 1548 cm^−1^. Exploring the spectra of the salivary glands tick tissues exposed overall quite similar features of those obtained for the gut ones. Concerning the protein amide, I-II region; the two peaks were observed at 1653 cm^−1^ and at 1548 cm^−1^. Figure 1 portrays the two averaged infrared absorption spectra of the two tissues; the gut and the salivary glands, with their peak assignment obtained by OMNIC^©^ software. Other features were detected those may also provide information on the molecular composition of both tissue types: (i) a peak at 1453 cm^−1^ was noticed for both of them which is associated to protein and lipid contributions arising from δ(CH_3_) and δ(CH_2_) and (ii) a peak 1237 cm^−1^ for the gut and at 1243 cm^−1^ for the salivary glands that may be attributed to the DNA ν_as_(PO_2_^−^). A separate section is designated for this spectral region.

**Figure 2:**
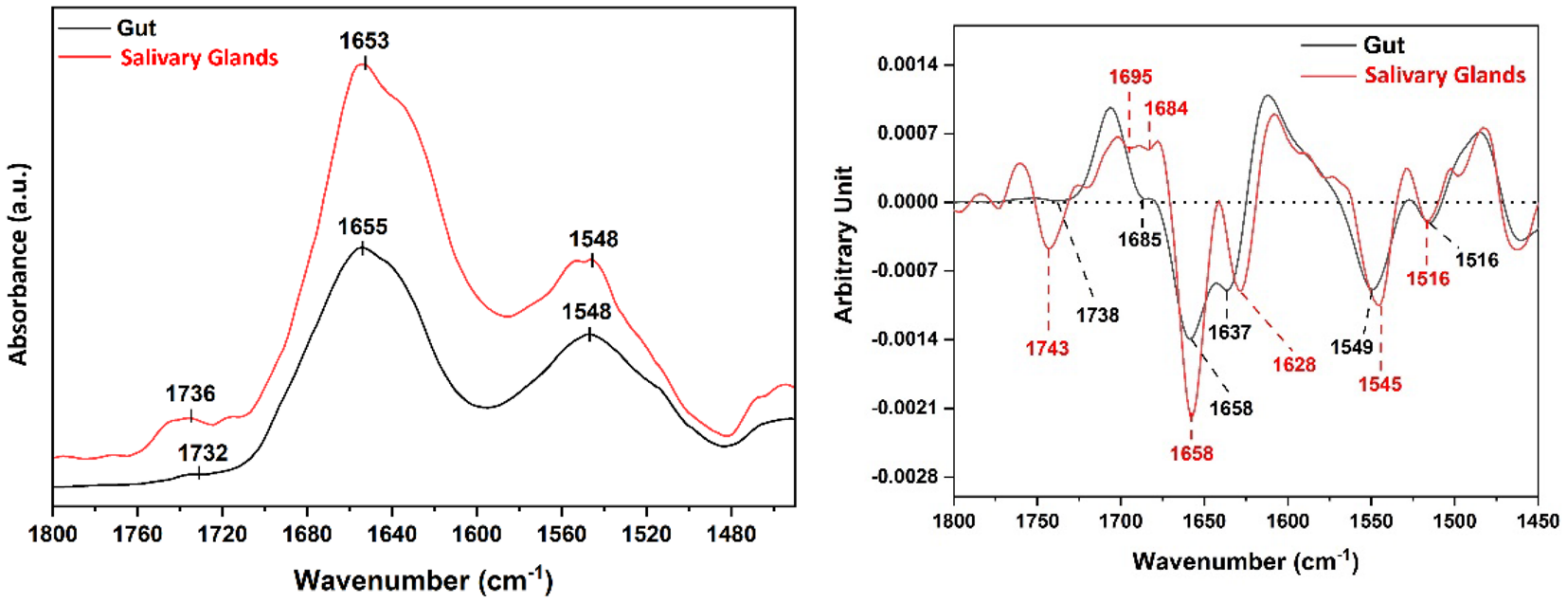
Average gut and salivary glands IR spectra showing different spectral features in the region (1800–1450 cm^−1^) (left). Their averaged and overlaid relative second derivatives (right).

**Figure 3:**
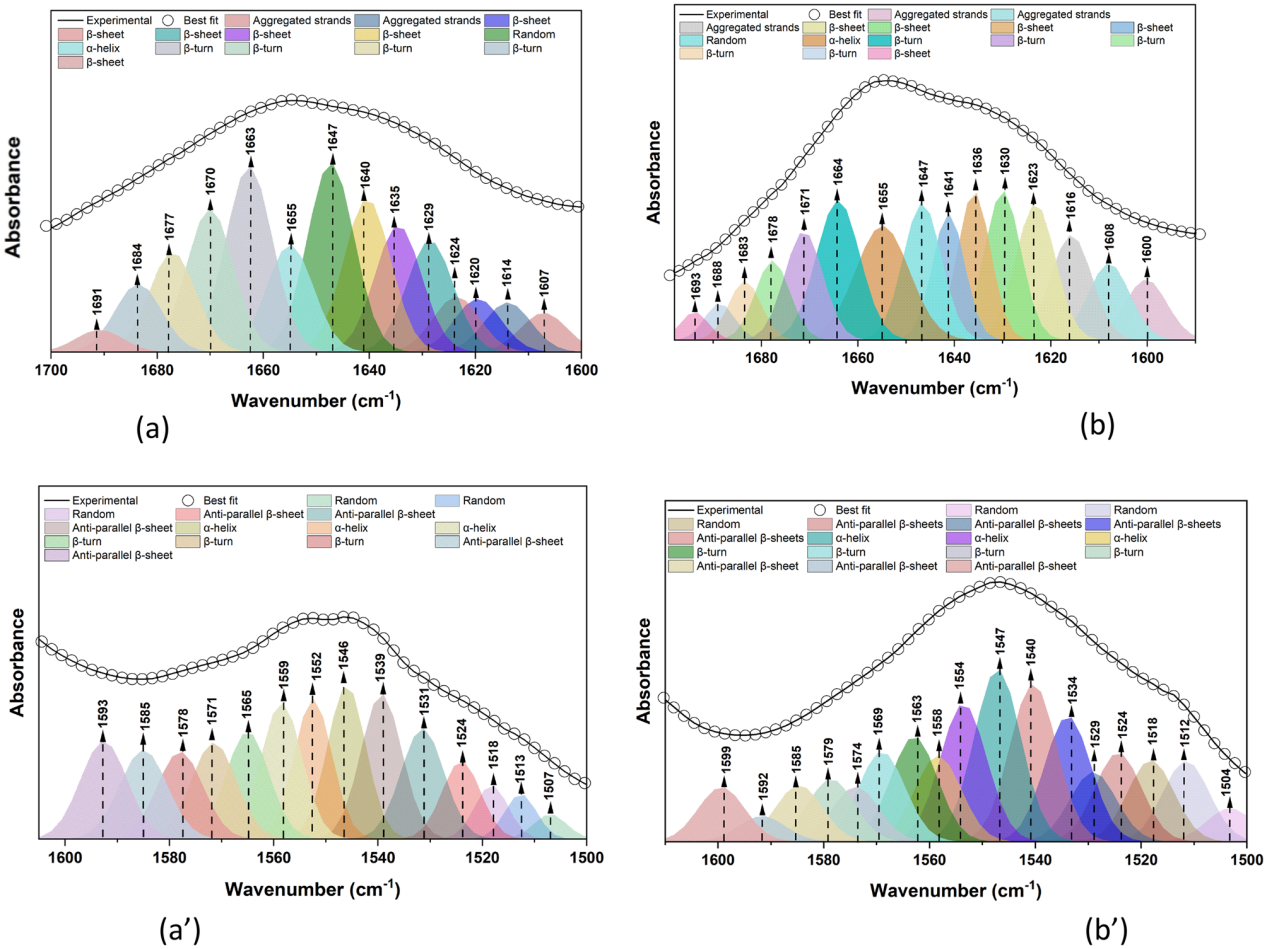
Amides I curve fitting of the gut (**a**) and salivary glands (**b**). In addition to amide II fitting for the gut tissues (**aʹ**), and the salivary glands ones (**bʹ**).

Precise information about the secondary structures’ contribution to proteins is very important to recognise structural changes of proteins. Amide I band (1600–1700 cm^−1^) is commonly considered the most sensitive spectral region that can be efficiently implemented in monitoring protein secondary structures^15^. In this study, second derivative methods were applied to both amides I and II to resolve the overlapped peaks such as α-helices, β-sheets, and β-turns of proteins. This in turn can shed some light on the conformational changes based on the proteins’ composition^16–19^. Figure 2 shows the average gut and salivary glands IR spectra showing their features in the 1800–1450 cm^−1^ region and their averaged and normalized second derivatives. We have also applied a curve fitting approach to the gut and the salivary glands tissue samples. Amide I, II bands for each of them were examined in order to obtain their protein conformations. Table 1 and Fig. 3 demonstrate the Amide I band deconvolution result of the gut, and the salivary glands, together with their associated secondary structures. A possible explanation for the higher ratio of aggregated strands in salivary gland and beta turn in gut tissues is that the tick’s salivary glands and gut have diverse functions, molecular compositions, and tissue characteristics, that facilitate their roles for the survival of the ticks which, ultimately depends on the secondary protein structures. Ixodid female’s salivary glands tissue has three different types of acini (I, II, and III). Type I acini which is the agranular anterior part connected to the main salivary duct, while type II and type III are the granular and glandular parts connected to the small and branched ducts. There’s no change in size of the type I acini, which is formed of epithelial, abluminal interstitial, and neck cells, during feeding. In contrast, Type II and III acini enlarge as feeding takes place, and have additional kinds of epithelium and various glandular cells enclosing the secretory granules. Additionally, a myoepithelial cell lining to the luminal surface of the type II and III acini occurs in a multi-folded web design. The fine structure of the cells facilitates the acini’s function, consisting of secreting and expeling saliva which in turn, facilitates acquisition of the blood during feeding. Simultaneously, it enables expulsion of about 75% of the blood meal containing unrequired fluid^20–22^. On the other hand, the crucial function of tick’s gut is receiving almost 100-fold of the host blood comparable to its original unfed weight. Therefore, gut epithelial cells are well adapted to the tick feeding habits, and to the process of digestion of the host blood. The luminal diameter and epithelial cell lining cells have different characteristics according to the stage of the blood feeding. In semi-replete females ixodid tick’s species, the generative epithelial cell is significantly enlarged and differentiated into mature digestive, phagocytic, and pinocytic cells. All the differentiated cells are adhered to the basal membrane cells. Additionally, the luminal diameter is expanded in its size as the feeding progresses^23,24^. Therefore, a high ratio of aggregated strands which are pleated with a lot of bends as a secondary structure, facilitates the function of the salivary glands’ organ. The protein’s beta turns seem to fit well with the functions of the gut, as, the beta turns secondary structure usually has a proline residue at the starting of the turn with less flexibility, and large amounts of glycine residues within the turn, providing greater flexibility for increasing gut’s size during feeding^25^.

**Table 1:** Gut versus salivary glands amide I secondary structures and their assignments.

	Centroid	Ratio (%)	Assignment
Gut tissues	1607, 1614, 1620	10	Aggregated strands
	1624, 1629, 1635, 1640	32	β-sheet
	1647	13	Random
	1655	8	α-helix
	1663, 1670, 1677, 1684	35	β-turn
	1691	1.5	Anti-parallel β-sheet

	Centroid	Ratio (%)	Assignment
Salivary glands tissues	1600, 1608, 1616, 1623	27	Aggregated strands
	1630, 1636, 1641	25	β-sheet
	1647	9	Random
	1655	11	α-helix
	1664, 1671, 1678, 1683, 1688	28	β-turn
	1693	1.1	Anti-parallel β-sheet

Amide II is not commonly subjected to the quantitative analysis of secondary structures possibly because protein composition sensitivity is decreased compared with Amide I, and therefore, it is less straightforward when dealing with complex biological tissues^26–29^. Amide II protein band is generally known to show less sensitivity towards the conformational changes of proteins compared to Amide I bands; therefore, we cannot conclude any biological significance at this stage of analysis. Amide I and II bands (1700–1500 cm^−1^) are significantly recognized in different IR spectral analyses in determining protein secondary structures and dynamics^28^. Amide II bands (1600-1500cm^−1^) are mainly derived from the in-plane NH bending vibrations (~ 40–60%), along with the CN stretching vibrations of around 18–40%. They are generally known to possess less sensitivity to predict protein conformation^29^, whereas Amide I band (1700–1600 cm^−1^) is mainly originating from the C=O stretching vibrations which constitute circa 80% of the peptide linkage of the amide groups, they are highly recognized for being the most sensitive entity to the slight variations in the molecular assembly^30^. With this, secondary structures, producing unique C=O stretching frequencies that are governed by their hydrogen bonding patterns, can consequently be assigned, and monitored. In addition, and due to the FTIR experimental limitations, a typical overlap between these patterns does result in single unresolvable Amide I absorbance maxima, which then requires mathematical methods to resolve the individual secondary structures. Our target here was to examine the amide II, aiming at fully investigating the amide regions, to extract all the possible information relevant to the protein structural components variations. Even though, we are presenting the obtained information on the secondary structure development of amide II bands for both tissue types through second derivative and curve fitting methods similarly to procedure performed for amide I. Information on the assigned Amide II secondary structures is shown in Table 2.

**Table 2:** Gut versus salivary glands Amide II secondary structures and their assignment.

	Centroid	Ratio (%)	Assignment
Gut tissues	1504, 1512, 1518	13	Random
	1524, 1529, 1534, 1540, 1585, 1592, 1599	39	Anti-parallel β-sheet
	1547, 1554, 1558	26	α-helix
	1563, 1569, 1574, 1579	21	β-turn

	Centroid	Ratio (%)	Assignment
Salivary glands tissues	1507, 1513, 1518	7	Random
	1524, 1531, 1539, 1585, 1593	40	Anti-parallel β-sheet
	1546, 1552, 1559	28	α-helix
	1565, 1571, 1578	23	β-turn

### Lipid spectral region (2800–3000 cm^−1^)

In our study, together with protein investigation, the lipids profile of the tissues was tracked in the spectral window 2800–3000 cm^−1^. For the gut tissue, lipid absorptions were observed at 2852 cm^−1^, 2871 cm^−1^, 2928 cm^−1^, and 2956 cm^−1^.

The peaks of interest for the salivary glands’ tissue, were found to be at 2854 cm^−1^, 2924 cm^−1^, and 2954 cm^−1^, due to antisymmetric and symmetric stretching of the long hydrocarbon chains related to lipids such as ceramides, phospholipids, or glycolipids^31,32^. In addition, lipid carbonyl stretching detecting the absorbance of carbonyl ester was distinguished at 1739 cm^−1^ as shown in Fig. 1 which was undetected in the case of the gut tissue samples.

**Figure 4:**
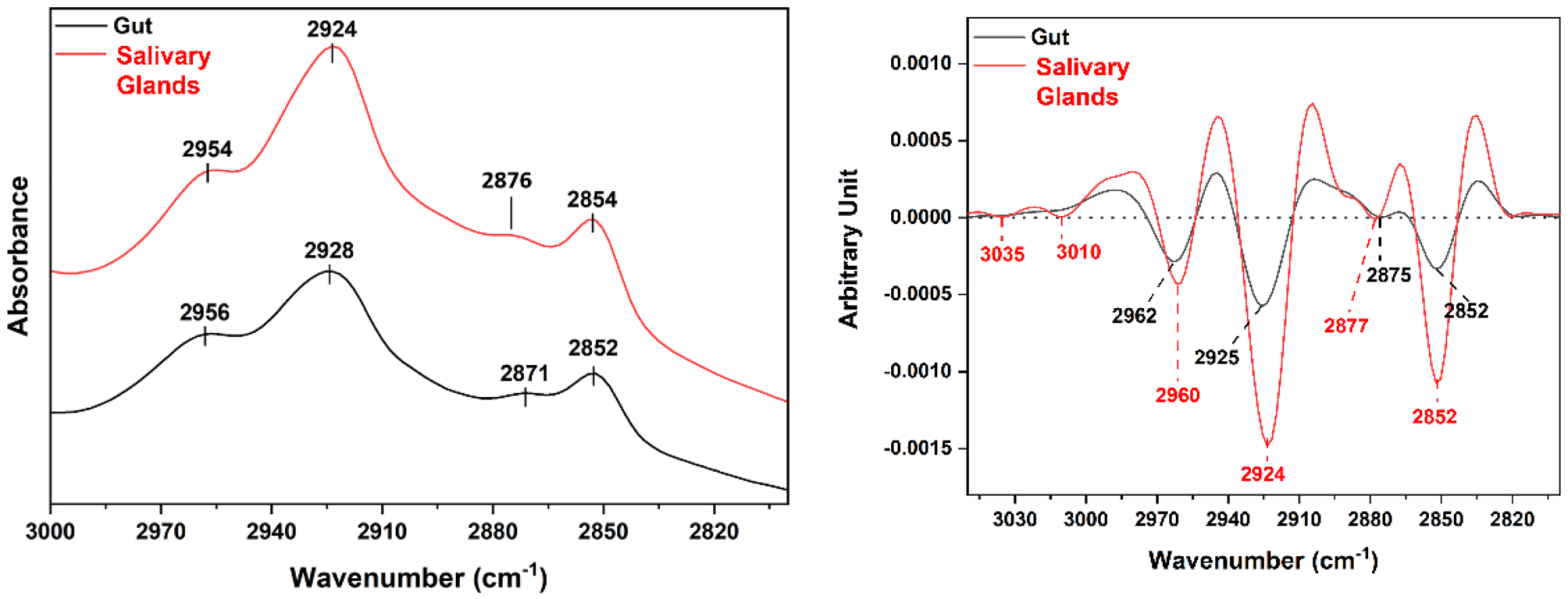
IR overlapped (common scale display for clarification) spectra of the lipid profile of the studied tissues in the 2800–3000 cm^−1^ region (left), their averaged second derivatives (right).

To obtain a better qualitative information on the contributing vibrations in both tissue types, second derivatives were calculated for this region (2800–3000 cm^−1^). The spectra and their second derivatives of both tissues are displayed in Fig. 4. More information on the peak positions is detailed in Table 3. A few differences were spotted between both tissues.

**Table 3:** Gut vs salivary glands lipid profiles peak assignment.

Average spectrum		Second derivative	
Gut	Salivary glands	Gut	Salivary glands
2852	2854	2852	2852
2871	2876	2875	2877
2928	2924	2925	2924
2956	2954	2962	2960

### Nucleic acid spectral region (1000–1500 cm^−1^)

**Figure 5:**
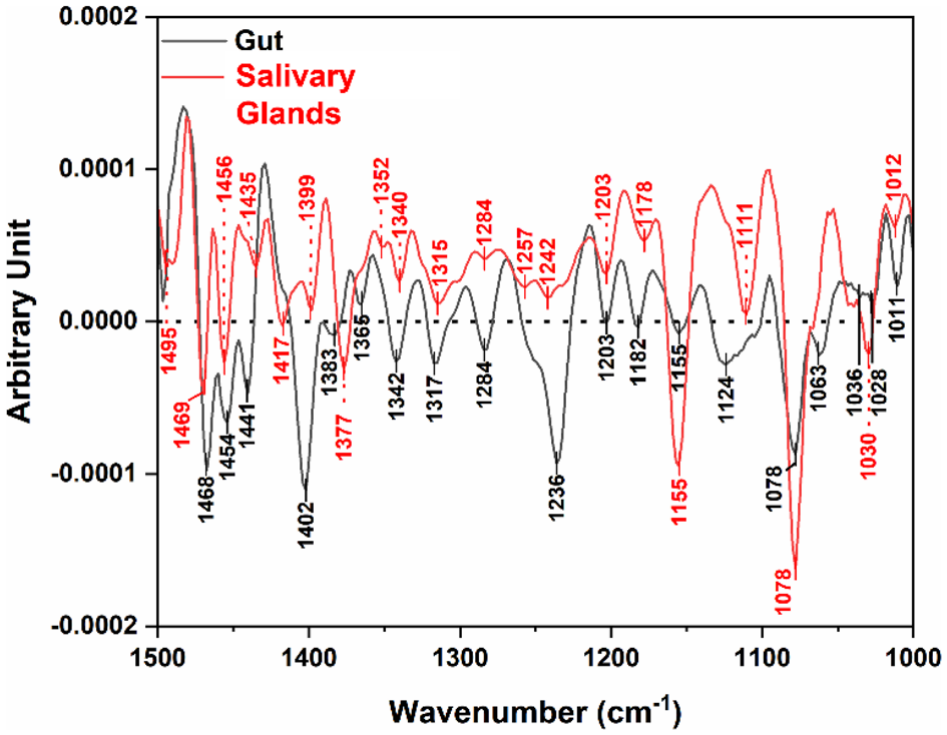
Second derivative comparative spectra of both investigated tissues.

Figure 5 demonstrates the second derivative spectra of the gut versus the salivary glands tissue samples in the 1000–1500 cm^−1^ spectral region. The major bands in this spectral region are mainly attributed to the vibrational modes of CH_2_ and CH_3_ bending and deformation (1500–1400 cm^−1^)^33^, and to the vibrational modes of carbohydrates and nucleic acids (1300–1000 cm^−1^)^30,34^. The bands at 1469 and 1468 cm^−1^ in the second derivative spectra of salivary glands and gut, respectively, are attributed to acyl chain CH_2_ bending and/or CH_3_ deformation, while those at 1456 and 1454 cm^−1^, as well as at 1435 and 1441 cm^−1^ in the second derivative spectra of salivary glands and gut, respectively, are attributed to CH_3_ deformation^33^. A band corresponding to –C–H, cis-deformation^33,35^ can be observed at 1417 cm^−1^ in the second derivative spectrum of the salivary glands but is not present in that of the gut.

The band at 1242 cm^−1^ in the second derivative spectrum of salivary glands and at 1236 cm^−1^ of gut represents the amide III coupled with asymmetric phosphodiester stretching mode (PO_2_^−^) from the nucleic acids^34,36^. The band at 1155 cm^−1^ in both spectra can be attributed to the C–O stretching vibration^37^ and the band at 1078 cm^−1^ in both spectra can be attributed to symmetric PO_2_^−^ stretching of phosphodiesters of nucleic acids^34,37^. Finally, the band at 1030 and 1028 cm^−1^ in the spectra of salivary glands and gut, respectively, is corresponding to the C–O stretching and C–O–H bending of carbohydrates^30,36,37^.

### IR chemical mapping

**Figure 6:**
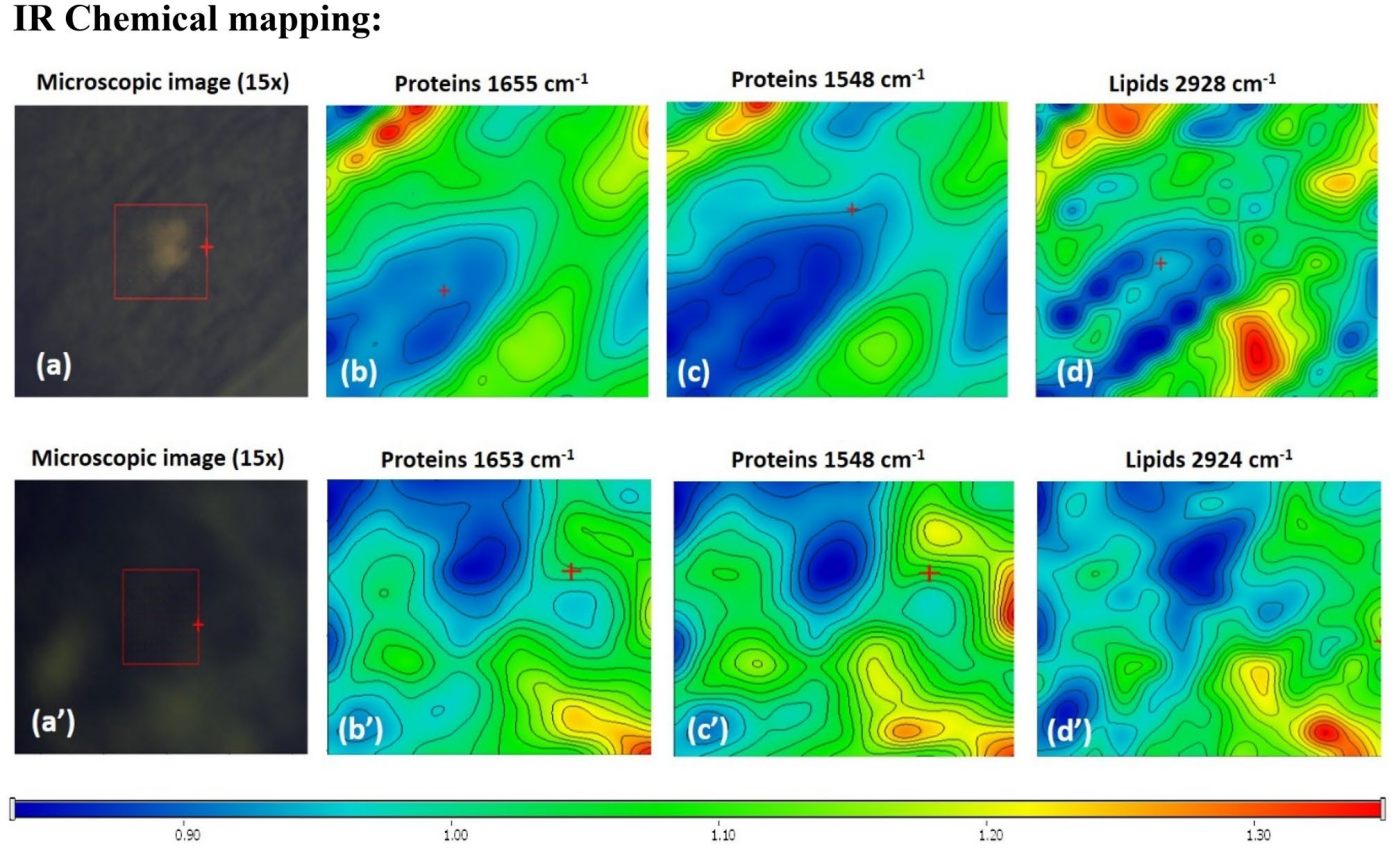
(**a**–**c**) are the chemical distribution of the proteins Amide I and Amide II bands, together with the lipids’ distribution related to the gut tissues, whereas (**aʹ**–**cʹ**) are that of the salivary glands tissue. All chemical maps are labelled at the shown intensity of each biomolecule.

The FTIR microspectroscopy method provided more insights on examined biological tissues of different levels of complexity. More information on the examined tissues was provided through the chemical IR maps (shown in Fig. 6) collected over a selected area offering spectral and spatial evidence on the biochemical distribution to be tracked. In addition to the obtained characteristic spectral bands, chemical maps depicting the spectral absorbance of specific wavenumbers (especially those representing the biochemical components of the biological sample) in color-coded images, in which red areas denote high signal intensity, while blue areas denote low signal intensity were also highlighted. Mapping also serves for further investigation of the distribution of the vibrational modes and visually clarifies the difference between the concentration and distribution of biochemical components present in different tissues (gut vs salivary glands).

For the gut tissue (Fig. 6a–c), the IR maps depict different points of interest (lipids (2928 cm^−1^), proteins amide I (1655 cm^−1^), amide II (1548 cm^−1^) bands). Whereas Fig. 5aʹ–cʹ also shows the infrared mapping of the lipids (2924 cm^−1^), proteins amide I (1653 cm^−1^), amide II (1548 cm^−1^) bands, for the salivary gland’s tissue. The IR chemical maps demonstrated a relatively close signal intensity between gut and salivary glands tissues in the protein's amide I region (a and aʹ), while in the protein's amide II region, the salivary glands tissue (bʹ) demonstrated a relatively higher intensity than gut tissue (b). Finally, the chemical maps of the lipids’ region demonstrated higher intensity in gut tissue (c) than in salivary glands tissue (cʹ). A possible explanation of this observation could be attributed to the fact that lipid is a crucial component of organelle specially cell membrane structures and important for signaling molecules such as hormones, enzymes, etc. Gut is the site where host blood uptakes, processed, and nutrients are absorbed which is essential for the survival and oviposition of the ticks^38^. Tick’s gut contains a network of acidic peptidases (enzymes) responsible for breaking down host blood proteins, including cysteine, aspartic, serine, and metallo-peptidases. Additionally, the enzymes required for hemogobinolysis, oxidative stress reduction and detoxification^39,40^. Moreover, midgut vitellogenic cells are involved in vitellogenin synthesis and processing which is crucial for ticks’ reproduction and initiated upon feeding begins^41,42^. Unfortunately, there’s no comprehensive study comparing tissue lipidomes in ticks. However, in some related taxa, Carvalho et al. investigated different tissues lipidomes in Drosophila. They stated that not all lipids are dominantly present in a subset of all tissues, however, some lipids are exclusively present in a subset of certain tissue according to the tissue function. For instance, hexosyl ceramides with hydroxylated fatty acid moieties are only found in the gut, and interestingly, these molecules are also enriched in the corresponding mammalian tissues, suggesting that their tissue-specific functions are conserved across phyla. Additionally, they observed tissue-specific lipids, with quantitative differences in the proportions of lipid classes among different tissues^43^. Hence, it will be interesting to also have a comprehensive study regarding lipidomes of tick’s tissue in the future.

### Median values of biochemical parameters

**Figure 7:**
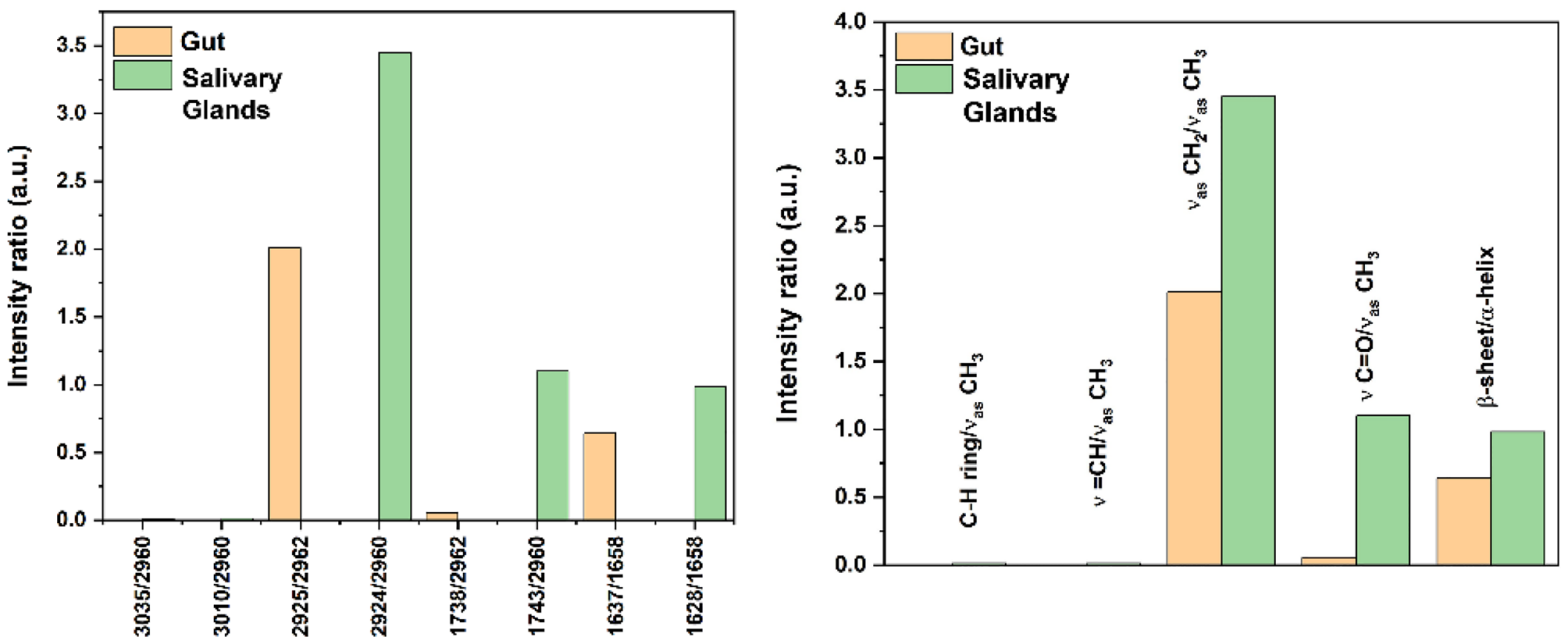
Absorbance ratios calculated for salivary glands and gut tissue types.

In order to gain extra indications about possible specific differences between both tissue types, especially those that may be hard to detect for semi-quantitative analysis, we also calculated the median values demonstrated in Fig. 7 comprising the peak intensity ratios calculations for chosen spectral bands. Again, using the second derivative intensities proved to be a very efficient method for guiding the studies of biomolecular assessments. Median values in the case of the salivary glands related to the lipids’ profile are chosen as 3010 cm^−1^/2960 cm^−1^, 2924 cm^−1^/2960 cm^−1^, and 1743 cm^−1^/2960 cm^−1^. On the other hand, for the case of the gut tissue, lipids’ profile chosen values were 2925cm^−1^/2962 cm^−1^, and 1738 cm^−1^/2962 cm^−1^.

As depicted in Fig. 4, some differences can be detected for both tissue types in the second derivatives of the lipid region examined between 2800 and 3050 cm^−1^. One feature that was followed is the unsaturated lipid peak (ν=CH) observed at 3010 cm^−1^, which is beneficial for examining the lipids profile unsaturation level^44^. That peak might be a result of the occurrence of lipid oxidation processes with the production of free radicals. This peak was observed for the salivary glands’ tissue with slightly detectable traces in the case of gut second derivative as can be seen in Fig. 4, where the intensity of the 3010 cm^−1^ peak is found to be relatively higher for the salivary glands second derivative spectrum. The ratio of 3010 cm^−1^/2960 cm^−1^ (ν(=CH)/ν_as_(CH_3_)) was calculated to get information on the unsaturation level. With this, we may suggest a higher degree of unsaturation, which in turn, may indicate a higher degree of oxidation of the lipids in the case of the salivary glands’ tissue compared to that of the gut’s tissue. This paradoxical finding could be due to the occurrence of innate nutritional immunity, which is one of the tick defense strategies^45,46^. During tick feeding period, they ingest huge amounts of blood which could reach 100 times of their unfed weight. Tick blood feeding habit could be toxic for the tick, as it contains a large amount of heme, iron, and other diverse other molecules which could promote oxidative stress and encounter high loads of free radicals^46,47^. Therefore, a maintained equilibrium is essential to detoxify the high iron and heme levels ingested during blood feeding. Ticks have heme-binding storage proteins such as vitelline (Vn), vitellogenin (Vg), and hemelipoglyco-carrier protein (CP) which is crucial for their survival, oviposition, and indirectly may have heme detoxification functions. In some tick species, for instance, *Dermacentor* (*D.*) *variabilis* and *D. marginatus* CP m-RNA were detected in salivary glands and weakly or not detected in guts^47–49^. Moreover, Vg production is initiated in the gut vitellogenic cells with the onset of feeding and then transported to the oocytes^42^. Vn could be considered as heme reservoir and can bind up to 30 molecules of heme in the oocytes, therefore the heme detoxification defense occurs with the onset of feeding and during embryonic development process^47,50^. Furthermore, iron equilibrium and regulation are maintained by ferritin (FER) proteins; FER1 and FER2. FER1 is iron storage protein and constitutively expressed in ticks’ gut while increased with the feeding in salivary glands. On the other hand, FER2 is iron transporter protein and its level in the gut is unchanged during blood feeding, however, it decreased in salivary glands with the progression of feeding^51^. Taken together, we could suggest that a balanced regulation of heme and iron levels and their encountered free radical is organ dependent therefore, our data showed higher level of unsaturated lipids in salivary gland more than gut tissues although the gut tissue is exposed to higher levels of heme and iron during ticks’ feeding period. The calculated absorbance ratios are illustrated in Table 4. As shown, the absorbance ratios of 3035 cm^−1^/2960 cm^−1^ and 3010 cm^−1^/2960 cm^−1^ were calculated only for the salivary glands and not for gut due to the absence of 3035 and 3010 cm^−1^ bands in the second derivative spectrum of gut. Otherwise, the rest of the absorbance ratios in the lipids’ region as well as that of beta-sheets/alpha helices in the protein region were calculated and compared.

**Table 4:** Absorbance ratios calculated for both tissue types.

Ratio	Gut	Salivary glands
3035/2960		0.012
3010/2960		0.013
2925/2962	2.01	
2924/2960		3.45
1738/2962	0.055	
1743/2960		1.10
1637/1658	0.64	
1628/1658		0.98

The change associated to the 2924 cm^−1^/2960cm^−1^ (salivary glands) and 2925 cm^−1^/2962 cm^−1^ (gut) corresponds to a change of ν_as_(CH_2_)/ν_as_(CH_3_). This ratio is known to correspond to possible conformational changes of lipid chains^33^. In our study, the median value of the 2924 cm^−1^/2960 cm^−1^ ratio for the salivary glands is 3.45, whereas, for the gut, it is found to be 2.01 (Table 4). Although the peaks’ assigned values are very close to each other, an increase in the ratio is found for the salivary gland’s tissues, which may suggest conformational changes in the order of lipid acyl chains and the existence of long fatty acids^44,52^.

The peaks observed at 1743 cm^−1^ (salivary glands) and at 1738 cm^−1^ (gut) are also indicative of lipid oxidation^44,53,54^. For both the cases 1743 cm^−1^/2960 cm^−1^ and 1738 cm^−1^/2962 cm^−1^, the calculated ratios (1.10 for salivary glands and 0.055 for gut) show that it is higher in case of the salivary glands when compared to the gut tissues indicating again an increase of the oxidation state of the lipids in the salivary glands’ biological tissues.

For the protein region, a chosen value of 1628 cm^−1^/1658 cm^−1^ was used for the salivary glands case, and 1637 cm^−1^/1658 cm^−1^ was associated to the protein absorption bands for the gut, to assess possible conformational changes. Slight conformational alterations were observed concerning the ratios of beta-sheets/alpha helices of both tissue types.

### Multivariate PCA analysis

In an attempt to introduce an additional approach aiming at validating the obtained spectral results. The Multivariate Statistical Analysis (MVA) was also applied to shed some light on the biochemical variations of both tissues under investigation. Using this statistical approach, in particular, the Principal Component Analysis algorithm, PCA, proves to be extremely advantageous. One of its important advantages is that it does not need any a *priori* information considering only the variance in the data, in addition to investigating multiple and numerous parameters in one step^55^ PCA calculates the best discriminating components contributing to the variance between samples of a given dataset which can be clearly visualized by the clustering of scores in the scores plot, while the exact components (i.e. wavenumbers, vibrational modes, biochemical components, etc.) contributing to this variance can be accurately obtained from the loading plots. The standard criterion of how the first PC (Principal Components) is chosen is that it contains most of the variance of the scores, with the subsequent PC contains less variance. Based on the original spectral characteristics, a score plot, reporting the score values of two different PCs for all the measured samples, allows visualizing, in a weighted approach, the similarities or the differences among the samples.

**Figure 8:**
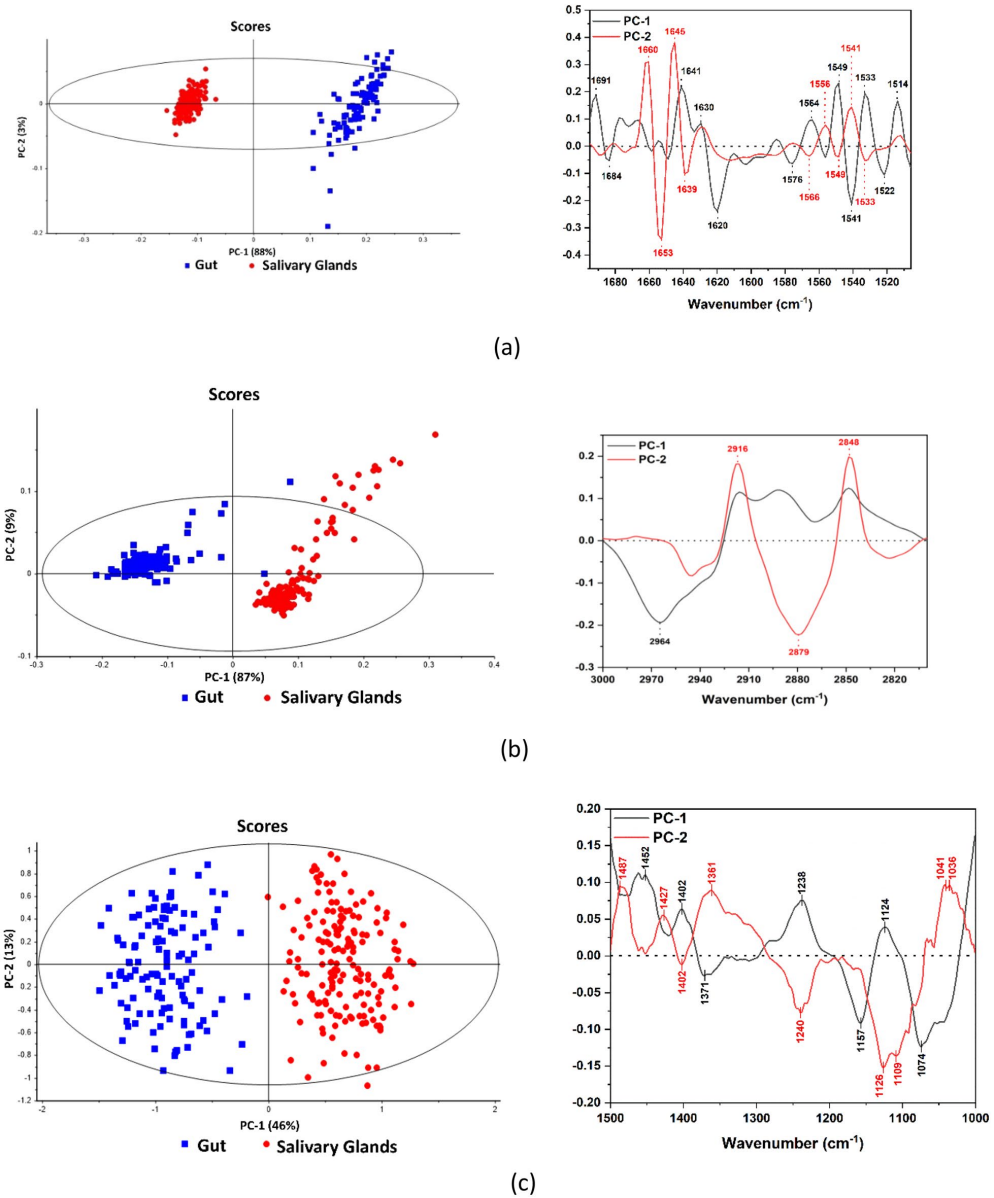
PCA score plots (left) and loading plots (right) for (**a**) proteins spectral region (1600–1700 cm^−1^), (**b**) lipids region (2800–3000 cm^−1^), and (**c**) the spectral region (1000–1500 cm^−1^).

The PCA score plots are shown in Fig. 8. The score plot of the amide region showed distinct separation between the scores of gut and salivary glands on PC1 with the scores of salivary glands falling in the positive part of PC1 and those of gut falling in its negative part. The scores of both groups show significant variation between the positive and negative parts of PC2. The plot clearly shows increased variation in the amide’s region within each group along both PC1 and PC2.

From a statistical point of view, the salivary glands scores (red dots) present positive values, whereas the gut tissue scores (blue squares) are characterized by negative values. Attempting to evaluating this PCA clustering result, it can be noted that this discrimination between the spectral information contained in both types of tissues develops because of the separation along the first component axis (46% of variance of PC-1) with the second component, PC-2, shows 13% of variance with the total variance of 59%. This statistical discrimination is considered to mainly occur because of the biological variations between the tissues in terms of the relative concentration of the proteins’ components as also suggested by their loadings’ spectra representing the PC-1 and PC-2 in the Amides’ region (depicted in Fig. 8a (right)) These results indicate the spectral region that strongly affects the clustering. The main secondary structures contributing to the variability along PC1 are those centered at 1684 cm^−1^ (β-turn), 1620 cm^−1^ (aggregated strands), 1576 cm^−1^ (β-turn), 1541–1 (α-helix) and 1522–1 (Anti-parallel β-sheet) having negative correlation with PC1, and at 1691 cm^−1^ (β-sheet), 1641 cm^−1^ and 1630 cm^−1^ (β-sheet), 1564 cm^−1^ (β-turn), 1549 cm^−1^ (α-helix), 1533 cm^−1^ (Anti-parallel β-sheet) and 1514 cm^−1^ (random) having positive correlation. On the other hand, the main secondary structures contributing to the variability along PC2 are centered at 1660 cm^−1^ (β-turn), 1645 cm^−1^ (random), and 1556 cm^−1^ and 1541 cm^−1^ (α-helix) having positive correlation, and at 1653 cm^−1^ (α-helix), 1639 cm^−1^ (β-sheet), 1566 cm^−1^ (β-turn), 1549 cm^−1^ (α-helix), 1533 cm^−1^ (Anti-parallel β-sheet) having negative correlation.

Similarly, for the lipids’ region, this region is recognized by profiling the lipids’ distribution in terms of its CH2 and CH3 symmetric and asymmetric stretching vibrations which can reveal vital chemical information. With a further examination of the tissues clustering, this region was also subjected to the PCA analysis to investigate any structural differences between the salivary glands and gut biological tissues. Figure 8b (left) depicts the scores plot of the lipids’ distribution. PCA model here demonstrated a total of 96% of variance with PC-1 and PC-2 of 87% and 9%, respectively. It also demonstrated once again that the salivary glands scores were clustered within positive values, whereas the gut scores were characterized by negative values revealing a “regional separation” of the lipids’ components.

The loading plot of the lipids’ region demonstrates the main vibrational bands contributing to the variability along PC1 and PC2, with the main band of PC1 centered at 2964 cm^−1^ representing CH_3_ asymmetric stretching, while the main bands of PC2 are centered at 2916 cm^−1^, 2879 cm^−1^ and 2848 cm^−1^ corresponding to CH_2_ asymmetric stretching, CH_3_ symmetric stretching and CH_2_ symmetric stretching.

The PCA scores plot of the 1500–1000 cm^−1^ spectral region of the spectra of gut and salivary glands as shown in Fig. 8c demonstrates that the model explained 91% of the variability in this region, with PC1 explaining most of the variability, 88%, and PC2 explaining 3%. The plot clearly shows the anti-correlation of the scores of gut and salivary glands along the PC1 axis, with the scores of salivary glands perfectly clustered in the negative part, while the scores of gut falling in the negative part. The loading plot of the 1500–1000 cm^−1^ spectral region indicated that the main vibrational bands contributing to the variability along PC1 are centered at 1452 cm^−1^, 1402 cm^−1^, 1238 cm^−1^ and 1124 cm^−1^, having a positive correlation with PC1, and at 1371 cm^−1^, 1157 cm^−1^ and 1074 cm^−1^, having negative correlation with it. On the other hand, the main bands contributing to the variability along PC2 and having a positive correlation with it are centered at 1487 cm^−1^, 1427 cm^−1^, 1361 cm^−1^, 1041 cm^−1^ and 1036 cm^−1^, while those having negative correlation are centered at 1402 cm^−1^, 1240 cm^−1^, 1126 cm^−1^ and 1109 cm^−1^.

## Conclusion

In this study, comparative biochemical analysis of *H. dromedarii* salivary glands and gut tissues were examined using SR-FTIR micro-spectroscopy qualitatively and semi-quantitatively at the molecular level. To the best of our knowledge, this is the first SR-FTIR microspectroscopy study ever performed on these tissues. Conformational analysis was carried out for the proteins and the lipids’ regions. This examination further proves the efficiency of the analytical approach used hereby for investigating and comparing complex biological tissues, yielding informative insights which can be consequently built on to advance the comprehensive understanding of such species, preferably with combining other analytical methods such as mass spectrometry (MS)-based techniques. In the omics era, MS-based techniques, including matrix-assisted laser desorption/ionization time-of-flight (MALDI-TOF) MS and liquid chromatography-tandem MS (LC–MS/MS) are considered important methods to scrutinize repertoires of proteins, transcripts, metabolites, and genes within the cells. The ability of these MS-based techniques for identification of such proteomes within cells require sample processing and preparation^56^. In contrast, the SR-FTIR micro-spectroscopy conformational analysis for the protein’s and the lipids’ regions of *H. dromedarii* salivary glands and gut tissues has been done without any special sample preparation. This agrees with Sheng et al. who investigate the chemical composition of mutant and wild types of *Caenorhabditis elegans* nematodes, which allow the detection of differences in the relative amounts of carbohydrates, proteins and lipids between both types through the vibrational spectroscopic spectra^13^. We concluded that the information gained from this analysis provides biochemically significant visualization of tick tissues, which may facilitate further identification of vaccine candidates for developing effective preventive tick control measures.

## Materials and methods

### Ethics

This study was approved by Medical Research Ethics Committee, National Research Centre, Egypt (No. 20188). All animal experiments were conducted in accordance with all institutional and national guidelines laid down by the International Animal Ethics Committee and in accordance with local laws and regulations. The authors complied with the ARRIVE guidelines.

### Tick rearing colony

One adult *H. dromedarii* (two-host tick species) full engorged female tick was collected from infested camel in Berqash market, Nahia, Giza, Egypt and identified according to Walker et al.^57^. It was maintained at 25 ± 1 °C with 75–80% relative humidity in the incubator for oviposition till hatching into larvae. One hundred-fifty hatched larvae had been raised on ears of 3 healthy New-Zealand white male rabbits weighing 1.5–2 kg using an ear bag technique. The collected fully fed nymphs had been incubated at 25 ± 1 °C with 75–80% relative humidity for molting into unfed adults. Three more rabbits were used to rear equal number of both unfed male and female adults (10:10 per animal) to obtain fully fed females. The 2nd generation of semi-fed adult females was obtained for tick dissection according to Al-Ahmed and Kheir^58^. The tick life cycle is maintained at the Tick Rearing Facility at the animal house, National Research Centre, Egypt. The rabbits had been housed and reared during the experiment in accordance with all animal ethics guidelines.

### Tick dissection

Tick dissection has been performed to get salivary glands and guts of semi-fed *H. dromedarii* adult females according to Patton et al. and Tidwell et al.^59,60^. Fifteen ticks were rinsed and submerged in double distilled water in a clean glass petri dish. Then, scutums were removed and salivary glands as well as guts were removed by pinching and pulling it out of the body cavity, washed in PBS, PH7 and preserved as three replicates of PBS and stored at − 20 °C. The samples were frozen for 7 days at − 20 °C from the time the organs were dissected until they were subjected to SR-FTIR chemical mapping. The salivary glands and gut tick tissues were preserved as whole organs due to their finest structure and thickness.

### SR-FTIR micro-spectroscopic measurements

Synchrotron FTIR microspectroscopy (SR-μFTIR) investigation was conducted at the BM02-IR beamline^61,62^ of SESAME (Synchrotron-light for Experimental Science and Applications in the Middle East), Jordan. Two to three sections were measured for each tissue type. Samples were deposited on CaF_2_ IR windows and an area of interest was selected to be chemically mapped and constructed with an aperture of 30 × 30 μm^2^ for each tissue section using Atlμs^©^ software (Thermo Fisher Scientific^©^, USA). Maps were collected in transmission mode using Thermo Nicolet 8700 FTIR spectrometer, coupled with a Nicolet Continuum infrared microscope (Thermo Fisher Scientific^©^, USA). The microscope is equipped with the single-point detector, Mercury Cadmium Telluride (MCT-A), a 10× visible objective, and a 15× [NA (Numerical Aperture) = 0.58] IR/visible Schwarzschild objective matched with a 15× condenser. Samples were mounted on a Prior Scan^©^ motorized sample stage and spectra were acquired with 256 co-added scans at a spectral resolution of 4 cm^−1^, levels Zero filling 1, and Happ-Genzel apodization window in the mid-IR range between 650 and 4000 cm^−1^ with Thermo OMNIC^©^ and Atlµs^©^ software package. Background spectra were collected to assess the spectral contribution. The background signal was measured from a part of the CaF_2_ substrate without biological tissue.

### SR-FTIR data spectral processing and statistical analysis

Spectral processing was applied to the raw data using OMNIC^©^ software (Thermo Fisher Scientific^©^, USA). Data were baseline automatically corrected and smoothed with 9 points of 2nd polynomial order using Gaussian smoothing method. Curve fitting and Savitzky-Golay’s second derivatives were performed using PeakFit^©^ V4.12 software (Copyright^©^ 2020 Systat Software, Inc.). The acquired IR area maps were split into their individual sets of spectra (144 spectra were collected for the gut tissue samples, and 169 spectra were collected for the salivary glands one). More than 100 spectra were extracted to analyze both tissue samples. The obtained noise level in the spectral window 2000–2200 cm^−1^ was given by RMS: 0.001 for the gut tissue map, and RMS: 0.003 for the salivary glands map). Each spectral region was baseline-corrected and smoothed with 5 points windows (2nd polynomial order) using Savitzky-Golay smoothing algorithm. The spectra of lipids were then unit-vector normalized, while those of the amide’s region were differentiated to the second derivative using Savitzky-Golay algorithm (3rd polynomial order) and 7 smoothing points. PCA was performed on the Unscrambler X 10.4 software (CAMO Analytics, Oslo, Norway) using NIPALS algorithm.

## Data Availability

All data generated or analyzed during this study is provided within the manuscript.
